# High-resolution characterization of CPD hotspot formation in human fibroblasts

**DOI:** 10.1093/nar/gkt912

**Published:** 2013-10-16

**Authors:** Anamaria G. Zavala, Robert T. Morris, John J. Wyrick, Michael J. Smerdon

**Affiliations:** Biochemistry and Biophysics, School of Molecular Biosciences, Washington State University, Pullman, Washington 99164-7520, USA

## Abstract

Repair of DNA lesions must occur within the chromatin landscape and is associated with alterations in histone modifications and nucleosome rearrangement. To directly associate these chromatin features with DNA damage and repair, it is necessary to be able to map DNA adducts. We have developed a cyclobutane pyrimidine dimer (CPD)-specific immunoprecipitation method and mapped ultraviolet damage hotspots across human chromosomes 1 and 6. CPD hotspots occur almost equally in genic and intergenic regions. However, these hotspots are significantly more prevalent adjacent to repeat elements, especially Alu repeats. Nucleosome mapping studies indicate that nucleosomes are consistently positioned at Alu elements where CPD hotspots form, but by 2 h post-irradiation, these same regions are significantly depleted of nucleosomes. These results indicate that nucleosomes associated with hotspots of CPD formation are readily rearranged, potentially making them accessible to DNA repair machinery. Our results represent the first chromosome scale map of ultraviolet-induced DNA lesions in the human genome, and reveal the sequence features and dynamic chromatin changes associated with CPD hotspots.

## INTRODUCTION

Exposure of cells to genotoxic agents, both chemical and physical in nature, is responsible for the initiation and promotion of the majority of human cancers. Despite the vast array of DNA-damaging agents, the single unifying principal is their ability to interact with cellular DNA producing potentially mutagenic and heritable lesions. DNA damage can result from interaction with endogenous factors, such as reactive oxygen species, as well as physical and chemical agents in the environment (1).

Ultraviolet (UV) radiation is the most common physical carcinogen in our environment and the major etiological factor in the development of skin cancer in humans. Cyclobutane pyrimidine dimers (CPDs) are the predominant lesions formed during UV exposure. UV-induced photoproducts form exclusively at dipyrimidines and preferentially at TT sites. CPD formation at dipyrimidines varies, with induction at TT>TC>CT>CC at a ratio of 55:33:11:1 (2). CPD formation is influenced by sequence context (3) as well as by chromatin environment (4).

The highly dynamic and condensed structure of chromatin is composed of nucleosomes separated by linker regions. The nucleosome core consists of 147 bp of DNA wrapped around an octameric core of histone proteins H2A, H2B, H3 and H4. Our laboratory has previously demonstrated a small bias in CPD formation in nucleosome linker regions compared with nucleosome core DNA (∼1.5–2-fold) [reviewed in (5)]. In addition, we have shown that CPD formation is highly influenced by the orientation of the DNA on the core histone surface, with CPDs forming at a 10.3 base periodicity (4). CPD formation in the core DNA is as much as 10-fold higher when the phosphate backbone is furthest from the histone surface compared with when it is in contact with the histones.

A cell’s ability to correctly repair DNA damage is essential for the continued vitality of virtually all organisms. Eukaryotic cells repair DNA lesions through two main pathways: base excision repair (BER) and nucleotide excision repair (NER). BER removes small non–helix-distorting damage to bases, for instance oxidation or methylation. NER is responsible for the removal of helix-distorting lesions in DNA such as CPDs. Xeroderma pigmentosum, a severe genetic disorder characterized by an exceptionally high incidence of UV-induced skin cancer, results from disruption of the NER pathway and indicates a crucial role for NER in preventing cancer development (6).

In eukaryotic cells, NER must take place within the complex landscape of chromatin. The presence of histones decreases the rate of repair of DNA damage compared with naked DNA by reducing the accessibility of damaged DNA to repair enzymes (7,8). However, the rate of CPD removal in *Xenopus* oocyte nuclear extracts is not influenced by the rotational setting of the adduct on the histone octamer (9). For efficient DNA repair to take place, nucleosome rearrangement must occur. Our laboratory and others have shown that NER is facilitated by histone modifications as well as by ATP-dependent chromatin remodeling complexes (10–12).

Determining histone modification states and nucleosome positioning at sites of DNA adduct formation will allow us to determine nucleosome level changes that may be related to repair initiation and progression. Changes in histone modifications and nucleosome positioning over the course of DNA damage and repair in human cells can be mapped using ChIP-chip or ChIP-seq techniques. However, without the corresponding map of DNA adducts it is impossible to determine which histone modifications may be associated with and potentially facilitate DNA damage recognition and repair. Therefore, it is necessary to develop a high resolution map of DNA adducts in human cells that can be correlated to nucleosome position and DNA sequence data to further characterize adduct formation. The ability to map DNA damage across the genome would provide an unprecedented snapshot of where CPDs form without bias toward genic and promoter regions, the typical targets of previous studies (10,13,14). In addition, such data would enable the identification of hotspots of CPD formation and determine the sequence and chromatin similarities that may permit increased lesion formation.

Strickland and Gentile (15) first described immunoprecipitation of UV-induced DNA adducts over two decades ago. More recently, Rochette *et al.* (16) used CPD-specific immunoprecipitation and polymerase chain reaction (PCR) to determine rates of CPD repair in telomeres. Teng *et al.* (17) used CPD immunoprecipitation (cpdIP) to map UV-induced DNA damage across the *Saccharomyces cerevisiae* genome. By probing microarrays containing 6256 probes spaced at an average of 290 bp covering the 12 Mb yeast genome, these authors were able to show CPD adduct formation and repair across the yeast genome.

We have developed a similar immunoprecipitation and microarray-based technique to map UV-induced DNA adducts within the human genome. We mapped CPDs across chromosomes 1 and 6 using high-resolution tiling arrays. These arrays cover 416 Mb of the human genome with 6.5 million probes spaced at an average of 35 bp. Mapping UV-induced DNA adducts at this high resolution has allowed us to determine sequence similarities where CPD hotspots form. In addition, we have determined nucleosome occupancy at regions of hotspot formation and changes in nucleosome occupancy at 2 h post-irradiation. This study not only gives new insight into DNA adduct formation in the human genome, but also advances our understanding of nucleosome level changes that occur shortly after DNA damage.

## MATERIALS AND METHODS

### DNA irradiation and fragmentation

Telomerase (tert)-immortalized normal human fibroblast monolayers (NHF1-tert) (18) were cultured in Dulbecco’s modified Eagle’s medium (Gibco Invitrogen) supplemented with 10% fetal bovine serum and 1% penicillin/streptomycin at 37°C in a humidified incubator at 10% CO_2_. DNA was isolated from unirradiated NHF1 cells using a previously described protocol (19,20) and irradiated to the indicated doses with 254-nm UVC. UVC was used to induce CPDs in this study as well as in most of the studies referenced. It is commonly used in laboratories despite the fact that UVC does not reach the earth’s surface. However, both UVC and UVB, which is more relevant to human exposure, efficiently form both CPDs and 6-4 photoproducts, the two most common UV-induced DNA lesions (21).

Ten micrograms of isolated DNA was diluted in 400 µl of 10 mM Tris-Cl (pH 8) and sonicated to an average fragment size of 300 bp using a Fisher dismembrator 500 fitted with a microtip and set at 40% power. Samples were sonicated for four cycles of 10 s on and 30 s off in ice water.

### Cell irradiation and repair

NHF1-tert cells were grown to confluence before UV irradiation. The media was replaced with phosphate buffered saline (PBS) before irradiating with 254-nm UVC to a dose of 12 J/m^2^ at room temperature. After irradiation, the cells were allowed to repair at 37°C in conditioned medium for the indicated period. Cells were harvested by scraping into PBS.

### CPD immunoprecipitation

Ten micrograms of αCPD antibody (clone TDM2, Cosmo Bio Co., Ltd, Japan) was bound to 100 µl of Protein G Dynabeads (Invitrogen) in 1× PBS with 0.5% bovine serum albumin overnight at 4°C while rotating. Before immunoprecipitation, sonicated samples were brought to final buffer conditions (1× PBS, 0.025% Triton X-100, 25 µg/ml yeast tRNA), boiled for 5 min and flash cooled in an ice water bath before adding the sample to the protein G-bound antibodies. This mixture was incubated at 4°C overnight while rotating. Beads were washed four times in wash buffer (1× PBS, 0.025% Triton X-100) before eluting at 65°C in TE with 1% sodium dodecyl sulphate. After treatment with RNase A (0.2 mg/ml) and proteinase K (0.2 mg/ml), DNA was ethanol precipitated and resuspended in 10 mM Tris, pH 8.0.

### Slot blot

Slot blots to monitor CPD enrichment in cpdIP pulldowns were performed as previously described (12). DNA was diluted in 0.4 N NaOH, boiled for 3 min and then diluted in 10× SSC (1.5 M NaCl, 0.15 M sodium citrate, pH 7.0). Samples were applied to nitrocellulose membrane (Bio-Rad) in triplicate wells using a slot blot apparatus (Bio-Rad) and immobilized by microwave treatment. The membrane was blocked with 5% non-fat dry milk before probing with the αCPD antibody followed by an HRP-labeled anti-mouse antibody (Bio-Rad). Blots were developed using ECL-Plus (Bio-Rad) and visualized on a STORM 840 Plus (GE Healthcare) in fluorescence mode (450 nm). Blots were stripped, and total DNA was assessed by reprobing with a ^32^P-labeled random-primed genomic probe and visualizing on the STORM 840 in phosphorimager mode. CPD signals were quantified using ImageQuant software and normalized to total DNA loaded per well.

### Ligation-mediated PCR

If samples were destined for microarray hybridization, sonicated fragments were blunt-ended using T4 DNA polymerase (20 min at 12°C). After ethanol precipitation the double-stranded DNA (dsDNA) was resuspended in double-distilled H_2_O, mixed with 2 µM asymmetrical linker (22) and T4 DNA ligase and incubated overnight at 16°C. This approach was chosen because DNA remained predominantly single stranded after immunoprecipitation (data not shown), and ligating linkers onto the sonicated dsDNA before immunoprecipitation significantly increased ligation efficiency.

CPD adducts are bulky and can block the polymerase during PCR amplification. Therefore, we removed CPDs by photoreactivation as previously described (23,24). Immunoprecipitated DNA was mixed with 30 uM photolyase in repair buffer (50 mm Tris-HCl, pH7.5, 100 mm NaCl, 1 mm ethylenediaminetetraacetic acid and 10 mm DTT) and irradiated at 365 nm using a Spectroline UV lamp (model ENF-240 C, Spectronics Corp., Westbury, NY) for 30 min through a Pyrex filter. Samples were PCR amplified as described using HotStart-IT Taq (USB) (22).

### Micrococcal nuclease (MNase) digestion

NHF1-tert cells were irradiated and collected as described earlier. Chromatin was digested as described (25). Briefly, isolated nuclei were resuspended in buffer (10 mM Tris, pH 7.4, 15 mM NaCl, 60 mM KCl, 0.15 mM spermine and 0.5 mM spermidine) and digested with 120 U/ml MNase (Affymetrix) in 1.5 mM CaCl_2_ for 10 min at 37°C. Reactions were stopped with 100 mM ethylenediaminetetraacetic acid and 10 mM ethylene glycol tetraacetic acid (pH 7.5). Following treatment with RNase A and proteinase K, samples were extracted with phenol chloroform and ethanol precipitated. Samples were run on a 1% agarose gel, and mononucleosome DNA was excised from the gel and recovered using Freeze N Squeeze DNA Gel Extraction Spin Columns (Qiagen).

### DNA labeling and microarray hybridization

CPD-specific pulldown and MNase samples were amplified, and uracil was incorporated using the Bioprime Array CGH Genomic Labeling System (Invitrogen). Samples were fragmented to ∼60 bp by treatment with uracil DNA glycosylase and labeled with biotin using the GeneChip WT Double-Stranded DNA Terminal Labeling Kit (Affymetrix) before probing Affymetrix GeneChip Human Tiling 2.0 R Array A microarrays. Kits and array probing were carried out according to manufacturers’ protocols.

### Tiling array analysis

CPD and MNase tiling array experiments were performed to identify regions of CPD formation and nucleosome occupancy on human (NCBIv36/hg18) chromosomes 1 and 6. Triplicate CPD data were collected for input samples and immunoprecipitated unirradiated (0 J_IP and 0 J_input) and irradiated (12 J_IP and 12 J_input) samples collected immediately after irradiation. MNase data represents unirradiated (0 J_MNase and 0 J_input) and 2 h post-irradiation (12 J_MNase and 12 J_input) duplicate samples. All sample probe intensities were normalized using the TileProbe (26) program, which is available with Cisgenome (27). TileProbe extends the popular tiling array normalization method MAT (28). TileProbe leverages the large amount of tiling array data in GEO to model probe effects, producing a standardized probe intensity that can be compared between experiments.

CPD Peaks were identified using the TileMapv2 program (default setting) (29). TileMapv2 calculated normalized log_2_ intensity ratios as well as a moving average log ratio (MA) for each probe comparing the CPD 12 J_IP and 0 J_IP samples. Individual probe intensity ratios were based on a weighted average of the surrounding 10 probes, 5 probes upstream and downstream (default settings). Putative CPD hotspots were identified as regions where the probe intensity of immunoprecipitated DNA from irradiated samples (12 J_IP) is significantly greater than a control immunoprecipitation from unirradiated samples (0 J_IP) (false discovery rate < 0.05). To confirm the existence of the CPD hotspots, we used the probe intensity ratios from the irradiated (12 J_IP/12 J_input) and unirradiated (0 J_IP/0 J_input) microarray experiments. Using the individual log_2_ probe ratio, an average log ratio for each potential CPD hotspot was calculated for the 0 J_IP/0 J_input and 12 J_IP/12 J_input experiments. If the peak’s 12 J_IP/12 J_input score was 3-fold greater than the 0 J_IP/0 J_input score and the 12 J_IP/12 J_input showed at least a 3-fold (log_2_ probe score ≥ 1.58) enrichment, then the region was defined as a CPD hotspot. The result of this analysis provided a set of CPD formation hotspots of high confidence.

Nucleosome occupancy was determined for unirradiated samples and 2 h post-irradiation. MNase digested samples were compared with undigested input samples using TileMapv2 (default settings). The nucleosome occupancies for the 12 J_MNase/12 J_input, 0 J_ MNase/0 J_input and 12 J_ MNase/0 J_ MNase datasets were calculated. The nucleosome occupancies of the CPD hotspots were calculated as the average of the log_2_ probe intensity ratios within each region.

### CPD repeat association

A repeat library for chromosomes 1 and 6 (RepeatMasker) was constructed using the data collected from the genome UCSC table browser version hg18. The association between CPD hotspots and repeat elements was determined by comparing the chromosomal location of LINEs, SINEs (Alu and non-Alu elements) and long terminal repeats (LTRs) to the location of the maximum MA score for the CPD hotspot. CPD and repeat elements were determined to be associated if a repeat element was located within 100 bp of the maximum MA score of a CPD hotspot. The frequency of associations between CPD hotspots and each class of repeat was calculated. To determine the association significance, we compared the frequency of observed CPD-repeat associations against the distribution of randomly generated data. Random data were generated using regions identical in size and number to the observed CPD hotspots. To ensure consistency between the observed CPD hotspots and the random regions, all random regions were selected from chromosomal segments containing sufficient probes to cover their lengths. One thousand random datasets were generated for each chromosome. Random regions were associated with repeat elements if they overlapped or if the distance between the repeat and random region was not >100 bp. The association frequency between random regions and repeat elements for each of the 1000 sets was calculated for each chromosome. Observed CPD hotspots were determined to be significantly associated with repeat elements if <5% of the random sets had association frequencies greater than the observed data (*P* < 0.05).

### qPCR validation

DNA from cpdIP was mixed with 0.72 µM of each primer and 2× Power SYBR Green PCR Master Mix (Applied Biosystems). Reactions were run on a StepOnePlus Real-Time PCR system (Applied Biosystems). Amplification consisted of heating the samples to 95°C for 10 min, then 35 cycles of 95°C for 15 s and 60°C for 1 min. Each amplification was performed in triplicate. Primer sequences were Hotspot 1: F(5′-AGTGGACTTGTTGGTGCATATTA-3′), R (5′-AGGAGAGGTTTGGAGTATGTTTG-3′), Hotspot 2: F (5′-CTGCGGAATGGG AGTAGAAAG-3′), R (5′-CTTGGAGGAACAGCTCAGAAG-3′), Neg 1: F(5′-CCCTGATAGGGATGACAATGAC-3′) and R(5′-GCCTCCTGGGAAAGTTGATAA-3′). Quantitation was performed by relative standard curve for both input and IP samples.

### PolyT repeat identification

Alu SINE elements were separated into two groups based on the detection of a poly A:T region near the 3′ end. These regions were identified for chromosomes 1 and 6 by searching for windows of 10 bp containing 90% T's. Overlapping windows were merged together to define chromosomal polyT regions. The set of chromosomal polyT regions was compared with the Alu repeat locations. Any Alu repeat that had a polyT region overlapping between the 50-bp sequence upstream to 20-bp downstream of the 3′ end was defined as a polyT Alu element. The 70-bp window around the 3′ end was selected to account for potential errors in transcription annotation of repeat elements.

### Classification of CPDs

CPDs were classified as genic or intergenic using the hg18 RefSeq genome annotation retrieved from the UCSC browser. This classification was based on the chromosomal location of the maximum MA intensity ratio associated with each CPD hotspot. If the maximum MA intensity ratio of a CPD hotspot was located within a transcribed region of a gene then it was defined as a genic CPD. Conversely, if the maximum MA score of a CPD hotspot was not located within a transcribed region of a gene then it was defined as an intergenic CPD. Intergenic CPDs were sub-classified as promoter associated if they were located within 2-kb upstream of the transcription start site of a gene. Genic CPD hotspots were defined as intronic if the maximum MA intensity ratio of the peak did not fall within the exon sequence of a gene. CPD hotspot enrichment for each class was determined by comparing the observed frequency with the genic and intergenic distribution for the 1000 random datasets.

### Nucleosome enrichment and depletion

Significant enrichment or depletions of nucleosome occupancy within the CPD hotspot regions relative to the entire genome were calculated to determine whether the CPD formation was associated with a change in local nucleosome occupancy. The average occupancy log_2_ intensity ratios of the unirradiated (0 J_MNase/0 J_input) and irradiated (12 J_MNase/12 J_input) MNase datasets were calculated for the identified CPD hotspot regions. These intensity ratios were used to calculate an average occupancy for the set of CPD regions on chromosomes 1 and 6. To determine the change in nucleosome occupancy induced by UV irradiation, we calculated the average occupancy log_2_ intensity ratios for the 12 J_MNase/0 J_MNase samples. Using a similar methodology as described earlier for determining a significant association between CPDs and repeat elements, we determined whether the observed average occupancy showed significant enrichment or depletion relative to random datasets. The average log_2_ intensity ratio for each of the 1000 random sample sets was calculated creating a distribution of log_2_ ratios for each chromosome. The random regions used in this analysis were the same as those used in the CPD-repeat association analysis. The absolute or the change in nucleosome occupancy was enriched if <5% of the random samples had an average log_2_ ratio greater than the observed data or depleted if <5% of the random samples had an average log_2_ ratio less than the observed data (*P* < 0.05).

## RESULTS

### Validating CPD-specific DNA immunoprecipitation

**Figure 1.**
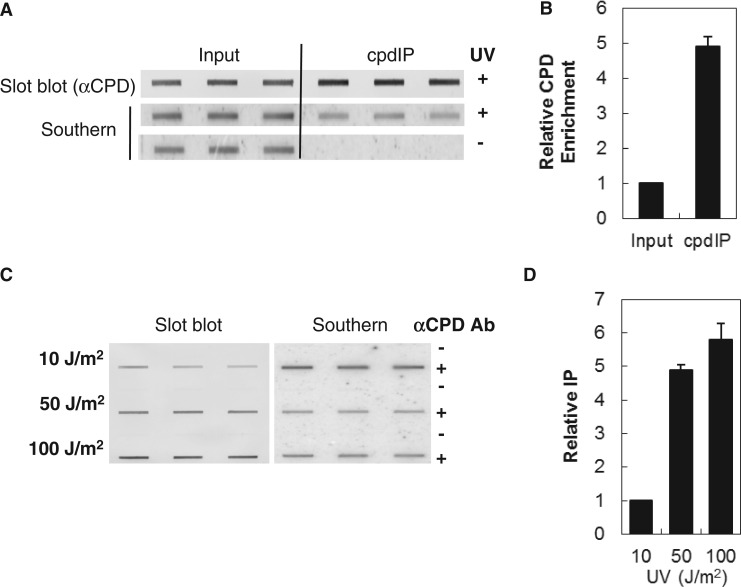
UV-specific DNA immunoprecipitation. (**A**) DNA was isolated from NHF1-tert cells and UV irradiated to 12 J/m^2^ before immunoprecipitation with CPD-specific monoclonal antibodies. Slot blots were probed with anti-CPD antibodies. Blots were stripped and reprobed with ^32^P-labeled genomic DNA to determine DNA loading. Slot blot signals were normalized against signals from the genomic Southerns. (**B**) Relative enrichment of CPD signal in immunoprecipitated samples. (**C**) Isolated DNA was irradiated with 10, 50 and 100 J/m^2^ UV before immunoprecipitation with (+) and without (−) CPD-specific antibody. Slot blots and signal normalization was performed as described earlier. (**D**) Relative enrichment of CPD signal in immunoprecipitated samples.

We mapped CPD lesions in the human genome using CPD-specific immunoprecipitation and high-resolution tiling arrays. To validate our IP protocol, we irradiated isolated genomic DNA at 12 J/m^2^ and fragmented the DNA to ∼300 bp by sonication. Because the CPD-specific antibody (clone TDM2) recognizes CPDs in single-stranded DNA, the fragmented DNA was denatured before IP. Following cpdIP samples were blotted to nitrocellulose and probed with an anti-CPD antibody to determine enrichment of CPD-specific DNA fragments compared with unprecipitated DNA (Figure 1A slot blot, +UV). To normalize the signal, the blot was stripped and reprobed with ^32^P-labeled random-primed genomic DNA to determine DNA loading (Figure 1A Southern +UV). We saw a 5-fold increase in CPDs in the immunoprecipitated samples compared with the input samples on slot blot analysis indicating an enrichment of DNA based on CPD recognition by the antibody (Figure 1B). The cpdIP pulldown is very specific, and nonspecific DNA was below the limit of detection by Southern blot in the pulldown fraction of unirradiated DNA (Figure 1A Southern −UV).

To ensure IP of irradiated DNA was dose dependent, isolated genomic DNA was irradiated to a final dose of 10, 50 and 100 J/m^2^. We quantitated the CPD content of samples immunoprecipitated with (+) and without (−) αCPD antibody by slot blot and normalized the signal for DNA loading as described earlier (Figure 1C). We saw a 5-fold increase in CPD pulldown at 50 J/m^2^ compared with 10 J/m^2^ (Figure 1D). However, we saw only a slight increase in CPD pulldown at 100 J/m^2^ compared with 50 J/m^2^. This finding may be due to some photoreversal of CPD lesions at this dose (30).

### Global mapping of CPD lesions in human chromosomes 1 and 6

We used CPD-specific immunoprecipitation to map CPD lesions in immortalized human fibroblasts following UV irradiation. Immunoprecipitated DNA fractions as well as input fractions were hybridized to separate Affymetrix GeneChip Human Tiling 2.0 R Array A. These microarrays contain >6.5 million 25-bp probes spaced at ∼35 nt that tile chromosomes 1 and 6. Genomic DNA was fragmented to an average size of 300 bp before immunoprecipitation; thus, CPD hotspots can be determined at a resolution of approximately this resolution. However, as the DNA fragments randomly distribute around the specific epitope recognized during immunoprecipitation, hybridization should be greatest for the CPD-associated probe compared with neighboring probes. The high resolution of the probes on the Affymetrix microarrays allowed us to more accurately identify specific sequences associated with CPD hotspot formation than we could have using microarrays with more distantly spaced probes. Repetitive elements, such as LINE, SINE and LTR, are not represented by probes on the tiling array, or are masked, to minimize cross-hybridization within these regions.

CPDs form at dipyrimidines across the entire genome; however, we were primarily interested in determining regions with the greatest amount of damage. Therefore, significant CPD hotspots were defined as probe hybridization signals of at least 3-fold (log_2_≥1.58) above input probe signals with the signal in the irradiated samples at least 3-fold greater than the signal in unirradiated control samples. This analysis yielded 1128 and 611 CPD-specific hotspots on chromosomes 1 and 6, respectively, in irradiated samples. The average maximum probe intensity for identified CPD hotspots was >6.0-fold (log_2_ = 2.6) above input on both chromosomes.

**Figure 2.**
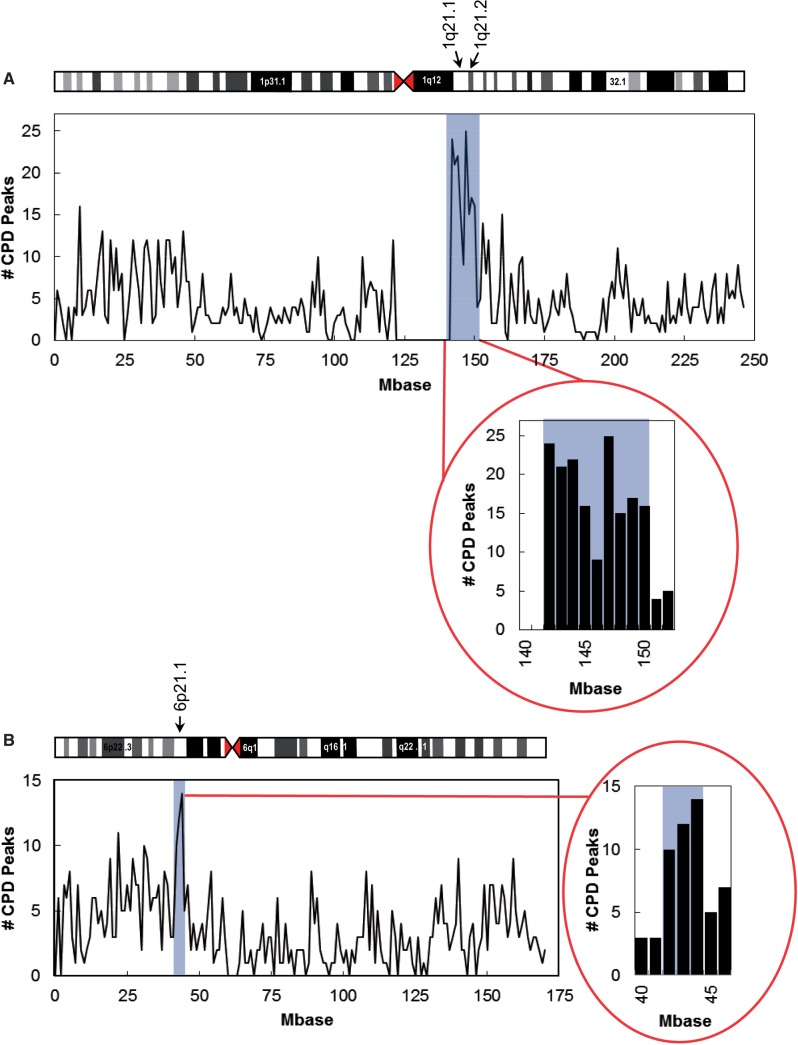
CPD hotspots mapped across chromosomes 1 and 6. NHF1-tert cells were UV irradiated at 12 J/m^2^. Isolated DNA was sheared and denatured before CPD-specific immunoprecipitation. Affymetrix Genechip tiling arrays 2.0 were probed with input and irradiated and unirradiated pulldown fractions. CPD hotspots were defined as peaks enriched at least 3-fold above input samples and at least 2-fold greater than unirradiated control samples. CPDs per megabase were mapped across (**A**) chromosome 1 and (**B**) chromosome 6.

If CPD hotspots were evenly distributed across the chromosomes, we would expect to see ∼4 CPD hotspots per megabase (Mb) (total number of CPD hotspots/chromosomal Mb) across both chromosomes. Figure 2A and B shows the concentration of CPD hotspots per megabase along chromosomes 1 and 6, respectively. The distribution of CPD hotspots does not appear to be random across either chromosome, instead showing distinct regions of increased density of hotspot formation. On chromosome 1, 165 CPD hotspots occur in the 9 Mb 1q21.1 and 1q21.2 region, including 45 hotspots in a 2-Mb intergenic region distal to the heterochromatic block (Figure 2A, inset). Although not quite as dramatic, we see a similar region on chromosome 6 where 36 CPD hotspots form within a 3-Mb region at 6p21 (Figure 2B, inset). Therefore, 14.6% of the CPD hotspots occur within ∼3.7% of chromosome 1 and 5.9% of CPD hotspots occur within ∼1.8% of chromosome 6. Interestingly, these regions of highest CPD density in chromosomes 1 and 6 correspond to replication-dependent histone clusters HIST1 at 6p21-p22 and HIST2 at 1q21 (31). In addition, these regions contain common fragile sites. Increased CPD formation at common fragile sites may be due to their unusual chromatin structure, including phased nucleosomes and increased CpG methylation [reviewed in (32)].

For simplicity, subsequent discussion will only highlight the individual chromosomes when significant differences occurred. The data for each individual chromosome are presented in the figures.

### CPD distribution in genic regions

**Figure 3.**
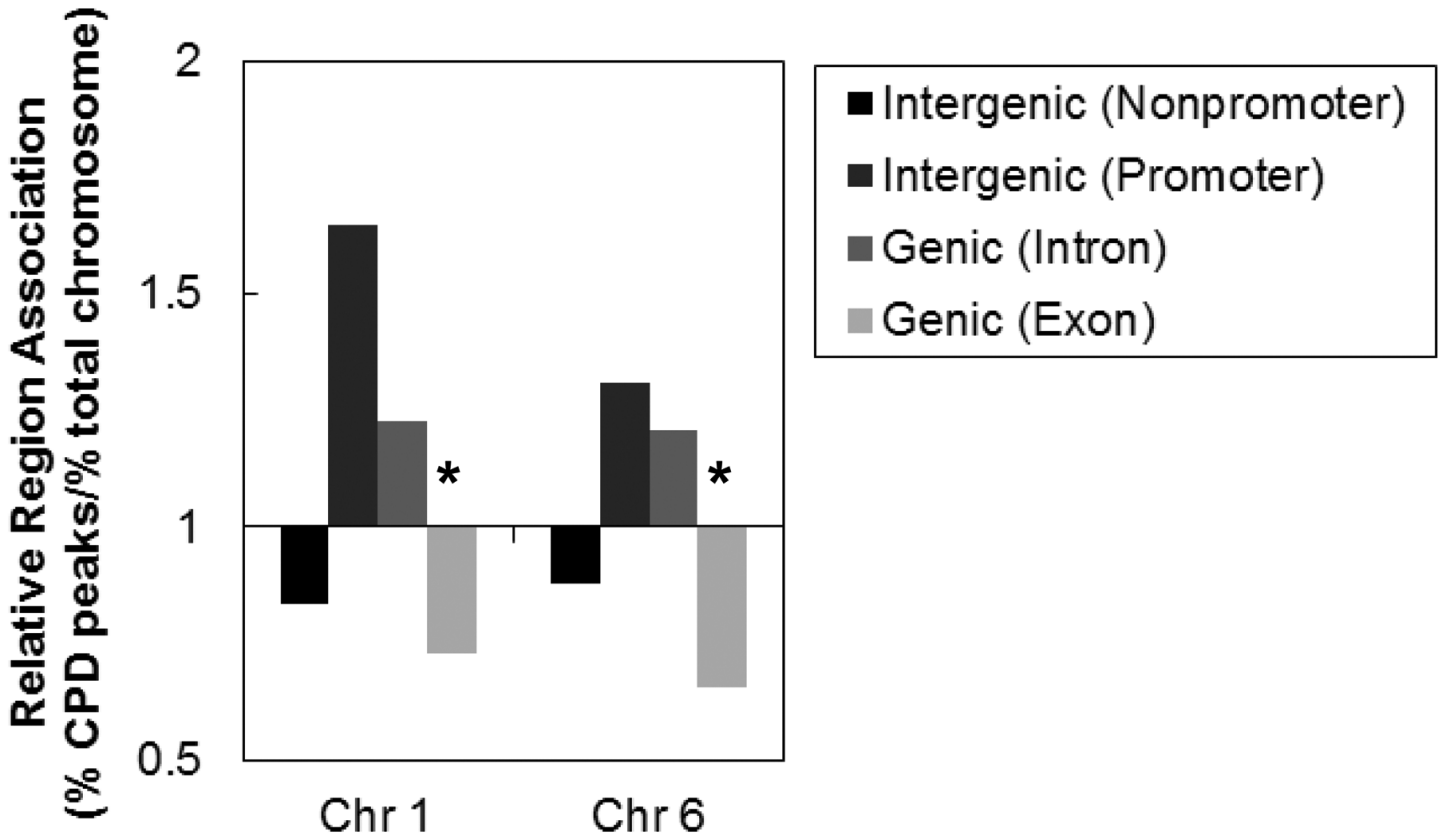
CPD hotspot association with genomic regions. CPD hotspot association with introns, exons and intergenic regions was determined based on NCBIv36/hg18 RefSeq genome annotation. Significant differences between observed and random datasets are denoted by *P < 0.001.

The distribution of CPD hotspots across chromosomes 1 and 6 was examined to determine whether CPD formation was biased toward genic or intergenic regions. CPD hotspots occurred almost equally in both genic (introns and exons) and intergenic (intergenic and promoter) regions, with 50.7% and 46.2% forming in genic regions and 49.3% and 53.8% forming in intergenic regions on chromosomes 1 and 6, respectively (Supplementary Figure S1). We normalized the distribution of CPD hotspots within intergenic, promoter, genic and exon regions to the distribution of these regions in the chromosome to determine whether there was any bias in formation (Figure 3). There was no significant CPD enrichment or depletion in intergenic (excluding promoter regions) and intron regions compared with random analyses. We defined intergenic regions within 2-kb upstream of a transcription start site to be promoter regions. Approximately 2% of CPD hotspots form within these regions, but this correlation is not significantly different compared with random associations (*P* > 0.1). However, CPD hotspots are significantly depleted in exon regions compared with random analyses (*P* < 0.001).

### CPD hotspots are associated with repeat elements

**Figure 4.**
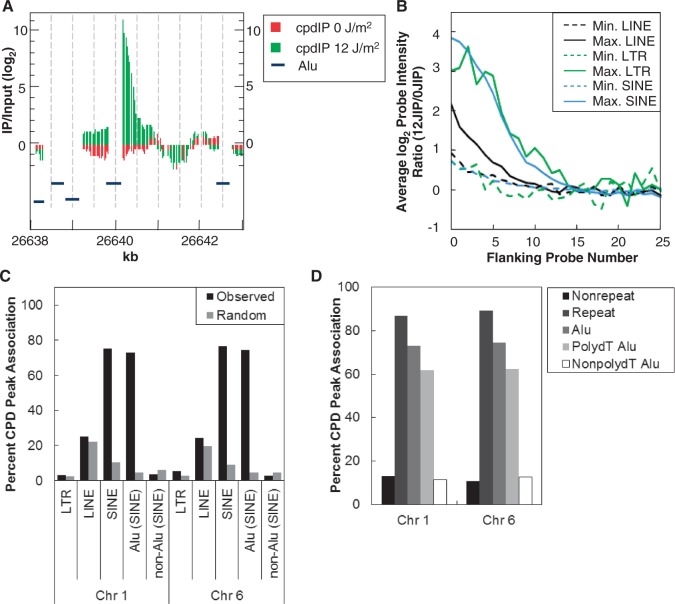
CPD hotspots form preferentially at repeat regions. (**A**) Representative association of CPD hotspot (green bars) compared with unirradiated DNA (red bars) at a repeat element on chromosome 6. Peaks were visualized using CisGenome software. Alu repeat elements are represented by blue bars. Gaps indicate masked repeat regions, which are not covered by probes. (**B**) CPD hotspot association with repeat elements was determined by comparing the chromosomal location of masked repeats (as determined by RepeatMasker) with the maximum MA score of identified CPD hotspots. CPD hotspots were determined to be associated with repeats if the maximum MA score of the CPD hotspot fell within 100 bp of the end of the masked repeat element. Average probe intensity at CPD hotspots was graphed for SINE (blue), LINE (black), and LTR (green) repeat elements. The edges of the masked repeat element are represented at 0 flanking probe number. Solid lines represent the side of the repeat with maximum probe intensity (Max.), whereas dashed lines correspond to the side of the repeat with lower probe intensity (Min.). Peak probe intensity of repeat elements significantly associated with CPD hotspots (SINE and LINE) consistently occurs unilaterally (Max.) within one probe length of the masked repeat. (**C**) CPD hotspot and random simulation associations with SINE, LINE, LTR and Alu repeat elements. Associations were determined to be significant compared with random simulations as described earlier. Significant differences are denoted by *P < 0.001. (**D**) CPD hotspot association at repeat elements and non-repetitive sequences. CPD hotspot association was further refined to characterize CPD hotspots associated with Alu repeats. Alu repeats were subdivided into polydT or non-polydT repeats. PolydT repeats contain 10-bp regions with at least 90% dTs.

The high resolution of the Affymetrix microarrays allowed us to further examine whether there were sequence similarities at the regions where CPD hotspots formed. We used the CisGenome (v1.2) genome browser to visualize the microarray probe intensity data (27). Interestingly, inspection of the data revealed that CPD hotspots frequently abutted masked repeat regions, which CisGenome displays as gaps in the probe data, indicating that CPDs may preferentially form within or near these repeats. Figure 4A is a representative CPD hotspot on chromosome 6 visualized in CisGenome. The green bars show the probe data as an MA, which yields a smoothed peak accounting for differences in probe hybridization based on sequence context. The maximum intensity ratio of the peak (based on the MA) is adjacent to a gap representing a masked repeat element. Blue horizontal bars show the location of Alu elements.

Repeat elements make up almost half of the human genome. We determined whether CPD hotspots associated with the most common repeat elements: LINE, SINE and LTR (33). CPD hotspots were determined to be associated with repeat elements if masked repeats were within 100 bp, three probes, of the maximum MA intensity ratio of a CPD hotspot. This analysis revealed that >86% of CPD hotspots were associated with LINE, SINE and LTR repeats. CPD hotspots were significantly enriched at repeat elements compared with random regions (*P* < 0.001). The average distance between a masked repeat and the maximum intensity ratio of a CPD hotspot (based on the MA) was less than one probe length, 25 bp, for LINE, SINE and LTR-associated CPD hotspots, indicating that CPD hotspots form either within or next to masked repeats (Figure 4B). For the majority of repeat-associated hotspots, the maximum probe intensity occurs unilaterally, i.e. on only one side of a masked repeat (Figure 4B, solid lines). The solid lines in Figure 4B represent the average log_2_ ratio of probes within the CPD hotspot adjacent to a repeat element (Max.), whereas the dashed line represent the average log_2_ ratio of probes on the opposite side of the repeat element (Min.). From these data we infer that the CPD lesions likely occur within, or immediately adjacent to, one end or side of the repeat element itself. Note that our data do not directly map the position of the CPD lesion within the repeat, as such elements are largely absent from the microarray, and would be difficult to measure directly. However, the hotspots detected in our dataset correspond to non-repetitive unique DNA sequences flanking the repeats, and thus are not likely to be confounded by the high copy number and the degree of sequence redundancy found in repeat elements.

Further refining where CPDs form at masked repeats shows that the majority of CPD hotspots form at SINE elements (∼76%), a greater than 7-fold enrichment over random predictions (*P* < 0.001) (Figure 4C). LINE and LTR elements are associated with ∼25% (*P* > 0.064) and 4% (*P* < 0.014) of CPD hotspots, respectively (Figure 4C). CPD hotspot association with LINE elements is not enriched over random predictions. Hotspot association with LTRs is slightly enriched, but only represents a small fraction of the repeat-associated hotspots. The total CPD to repeat association is >100% because ∼20% of repeats are associated with more than one repeat element (Supplementary Figure S2).

CPD hotspots associated with repeat elements are distributed approximately evenly between genic and intergenic regions (Supplementary Figure S3). CPD hotspots are enriched an average of 7-fold at repeat elements in both genic and intergenic regions compared with CPD hotspots not associated with repeats. The association of CPD hotspots with repeat regions is significantly enriched compared with random associations (*P* < 0.001).

Alu elements are the most abundant SINE, making up ∼10% of the human genome. More than 73% of CPD hotspots are associated with Alu elements, a greater than 10-fold enrichment over random predictions (*P* < 0.001) (Figure 4C).

The correlation coefficients for CPD hotspots per Mb compared with Alu elements per Mb are 0.550 and 0.712 for chromosomes 1 and 6, respectively, indicating a positive correlation for CPD hotspots to Alu elements (Supplementary Figure S4). The correlation coefficients for random regions per Mb compared with Alu elements per Mb are 0.072 and −0.142 for chromosomes 1 and 6, respectively, indicating a weak or negative correlation for random regions to Alu elements. CPD hotspots are depleted at non-Alu SINE repeats compared with random predictions (*P* ≥ 0.02) (Figure 4C). Because CPD hotspots appear to be most significantly associated with Alu repeat elements, we focused on further characterizing this association.

### CPD formation at polydT tracts

PolydT tracts are the most common repetitive elements in the human genome, primarily due to their occurrence in LINE and SINE retrotransposons (34). CPDs form preferentially at TT dinucleotides (3,35); therefore, increased formation within repeat regions could be due to polydA tracts at the 3′ end of retrotransposon insertion sites. Because polydT tracts >10 bp are overrepresented in eukaryotes, including *S. cerevisiae* and humans (36), we scanned the sequence for 10-bp windows containing at least 9 dTs. The identified polydT tracts had an average length of 14 bp. PolydT tracts were further characterized to determine whether they occurred at Alu repeat elements. Alu repeats were termed polydT associated if the 10-bp polydT region occurred within 50 bp of the end of the masked repeat or within 20 bp outside the masked region.

More than 73% of Alu-associated CPD hotspots are associated with polydT repeats (Figure 4D). However, only 50% of those hotspots are within 100 bp of the polydT tract (*P* < 0.001) (Supplementary Figure S5). This is noteworthy because the association of CPD hotspots with these repeat elements may not be solely due to long polydT stretches associated with Alu SINEs. If all of the CPD hotspots formed at polydT tracts, we could propose that sequence is the most important factor in CPD hotspot formation. However, the observed correlation indicates that factors other than sequence may influence CPD hotspot formation. Clearly, one such factor could be chromatin structure within these regions.

**Figure 5.**
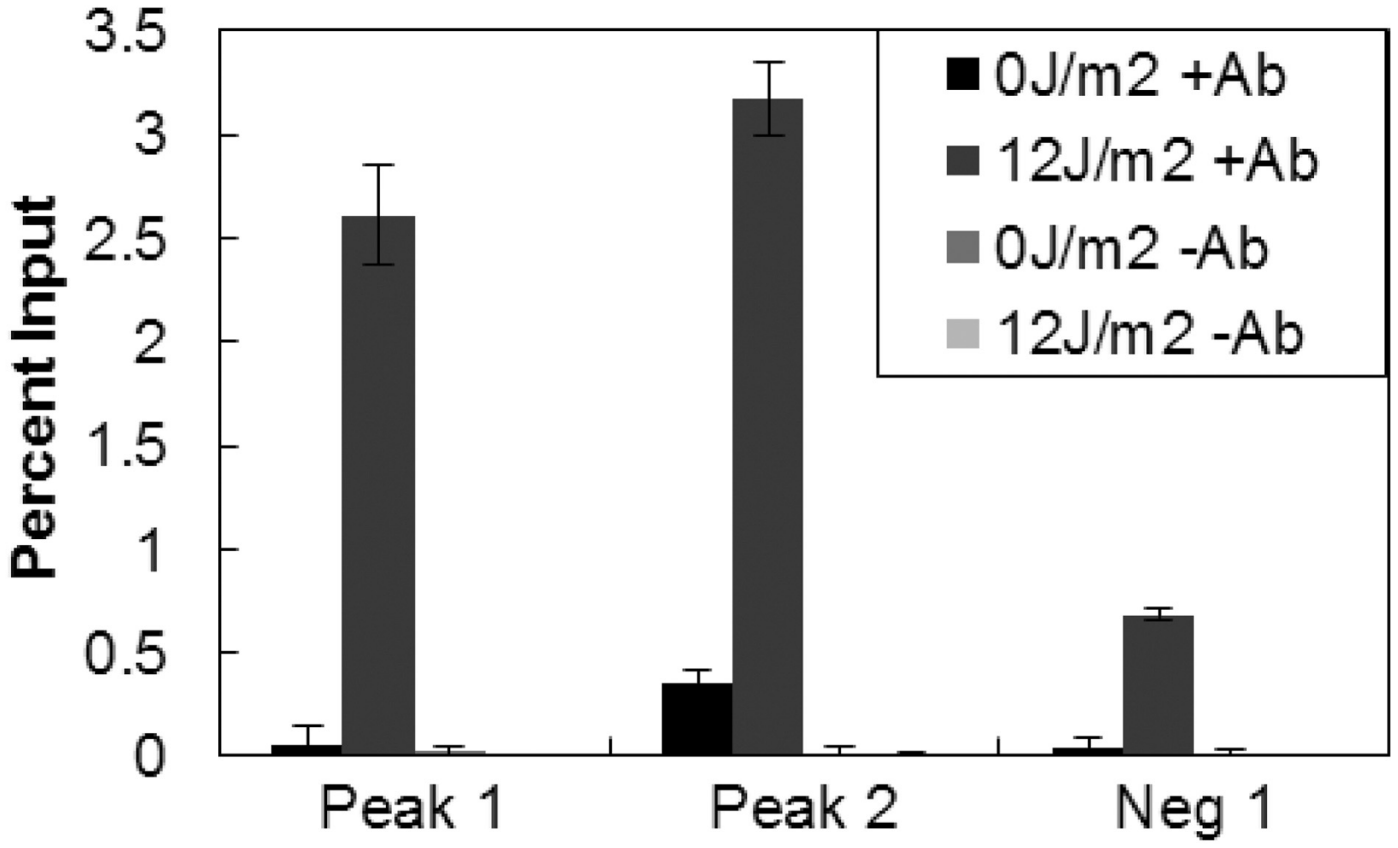
qPCR confirmation of identified CPD hotspots. Microarray-based CPD association with Alu repeats was confirmed by qPCR for regions associated with hotspots and a region not associated with a hotspot. Percentage input was determined for IPs of irradiated and unirradiated samples with and without CPD-specific antibody.

We also performed qPCR to confirm that the identified CPD hotspots were not simply due to sequence biases of the microarrays. Amplification of regions both associated and not associated with identified CPD hotspots confirmed the microarray results (Figure 5, Supplementary Figure S4). CPD-specific IPs of UV-irradiated samples were enriched at CPD hotspots more than 9-fold over unirradiated samples. Because CPDs do form at lower levels at dipyrimidines across the genome, and not simply at the identified hotspots, we also saw amplification at a region not associated with a CPD hotspot in UV-irradiated samples, but not in unirradiated samples. However, amplification in the non–hotspot-associated regions was almost 4-fold less than at identified CPD hotspots.

### Nucleosome occupancy at CPD hotspots

Nucleosome positioning is affected by DNA sequence (37,38), and Alu elements are strong nucleosome positioning sequences within the human genome (39). Regions of ∼100 bp immediately upstream and downstream of Alu elements are depleted of nucleosomes with phased nucleosomes extending in both directions. The phasing becomes weaker as distance from the Alu elements increases (39).

**Figure 6.**
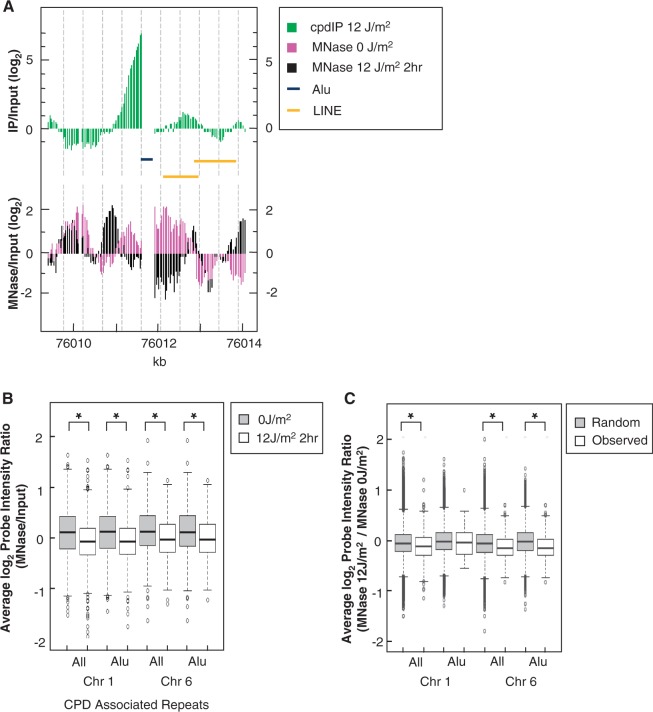
Nucleosome occupancy and remodeling at CPD hotspots. NHF1 cells were collected immediately before irradiation (0 J/m^2^; 0 JIP/0 Jinput) or irradiated with 12 J/m^2^ UV and collected at 2 h post-irradiation (12 J/m^2^; 12 JIP/12 Jinput). Chromatin was MNase digested and mononucleosome DNA was isolated and used to probe microarrays. (**A**) Representative CisGenome image of a CPD hotspot (green), nucleosome occupancy immediately before irradiation (pink) and nucleosome occupancy after 2 h of repair (black) on chromosome 6. Alu and LINE repeat elements are represented by blue and yellow bars, respectively. (**B**) Average nucleosome occupancy probe intensity ratios (log_2_) were calculated for the identified CPD hotspots associated with repeat elements for unirradiated (0 J/m^2^ IP/0 J input) and irradiated (12 J/m^2^ IP/12 J input) MNase datasets. Significant differences (*) between the nucleosome occupancy of the 0 J/m^2^ and 12 J/m^2^ samples were identified (Wilcoxon rank-sum test; *P* < 1 e-9). (**C**) Change in nucleosome occupancy probe intensity ratios (log_2_) was calculated for identified CPD hotspots and random regions associated with repeat elements in unirradiated and irradiated samples (MNase 12 J/m^2^/MNase 0 J/m^2^). Significant differences (*) between the nucleosome occupancy of the 0 J/m^2^ and 12 J/m^2^ samples were identified (Wilcoxon rank-sum test; *P* < 1 e-9).

We determined nucleosome occupancy within our identified CPD hotspots to see whether positioned nucleosomes might influence CPD formation in these regions. Unirradiated NHF1-tert cells were harvested and the DNA was MNase digested. Isolated mononucleosome DNA was fragmented to ∼60 bp before probing Affymetrix human tiling arrays. We performed duplicate hybridizations with biological replicates of MNase-digested DNA and input DNA to determine nucleosome occupancy across chromosomes 1 and 6. Using CisGenome, we saw that the MNase probe intensity ratios, which are directly proportional to the level of nucleosome occupancy and positioning, were higher at CPD hotspots than in surrounding regions (Figure 6A, pink bars). Average nucleosome occupancy at all repeat-associated CPD hotspots as well as at Alu-associated CPD hotspots was enriched compared with random simulations (*P* < 0.03) (Figure 6B).

Nucleosomes are inhibitory to DNA repair and must be rearranged or displaced for efficient repair to occur (5,7,40). Therefore, we wondered if we could see a change in nucleosome occupancy at CPD hotspots that would indicate the initiation of DNA repair. We mapped nucleosome occupancy, as described earlier, in irradiated NHF1-tert cells that had been allowed to repair for 2 h. Intriguingly, we saw that nucleosome occupancy decreased by 2 h post-irradiation (Figure 6A, black bars). On chromosome 6, average nucleosome occupancy at all CPD-associated repeat elements, including Alu elements, was significantly depleted at 2 h after irradiation compared with random associations (*P* < 0.022) (Figure 6C). On chromosome 1, nucleosome occupancy was significantly depleted among all CPD-associated repeat elements (*P* < 0.001), but was not significantly different from random simulations at Alu element-associated CPD hotspots (*P* > 0.39) at 2 h after irradiation (Figure 6C). However, average nucleosome occupancy at Alu-associated CPD hotspots was significantly depleted by 2 h after irradiation (12 J_MNase/12 J input) compared with nucleosome occupancy before irradiation (0 J_MNase/0 J input) on both chromosomes (*P* < 0.001) (Figure 6B). Depletion of nucleosome occupancy at CPD hotspots after irradiation indicates DNA damage-induced nucleosome rearrangement.

## DISCUSSION

Numerous studies have looked at CPD formation and repair in defined genes or promoter regions [e.g. (41–43)]. We sought to characterize CPD formation across the human genome using microarray technology. To our knowledge, this is the first report of tiling array analysis of CPD formation in the human genome. Using microarray analysis removed the bias of determining CPD formation strictly within a defined region of interest, allowing for a more comprehensive understanding of CPD adduct induction. We were particularly interested in characterizing CPD hotspot formation.

The first report of genome-wide mapping of CPDs was done in *S. cerevisiae* at an average resolution of 290 bp (17). These authors validated that CPD-specific antibody pulldown could be used to map CPD formation and repair across a genome. In the present study, we mapped CPD hotspots across chromosomes 1 and 6. Unexpectedly, we show a strong correlation between CPD hotspots and masked Alu elements. In contrast to the human genome, where repeat elements account for 50% of the genome, repetitive DNA makes up only 3–5% of the *S. cerevisiae* genome (44). This might explain why the previous study by Teng *et al*, did not observe the same striking association of CPD hotspots with repeat elements.

Although repeat sequences are frequently used to assess mismatch repair (45,46), there have been few studies of CPD formation and NER within Alu repeats. Englander and Howard (47) showed that CPDs formed preferentially at discrete sites within Alu repeats and were efficiently repaired. However, this report suggested using the repetitive nature of the Alu elements as a proxy to assess global adduct formation and repair. We show that, at least at the dose of UV irradiation used, there is increased CPD formation at a limited subset of Alu elements and therefore would not be representative of total genomic damage.

In this study, we show that CPD hotspots are not randomly distributed within the human genome, instead forming preferentially at Alu repeat elements. These repeat elements not only have distinctive sequence features, but also confer a unique chromatin environment. Nucleosomes are preferentially positioned within Alu elements with phased nucleosomes radiating out from the repeat element (39). The positioned nucleosomes are surrounded by ∼100-bp linker regions. We have confirmed that nucleosome occupancy is enriched at Alu elements where CPD hotspots form, indicating strong nucleosome positioning. In addition, many Alu elements contain polydA:dT tracts. PolydA:dT tracts form rigid structures that resist nucleosome formation (48–51). Nucleosome depletion extends beyond the border of the polydT regions up to 150 bp (49). The unique structure of these regions of DNA may make them more accessible not only to DNA adduct formation, but also to DNA binding proteins, such as those involved in DNA damage recognition. Several studies have suggested that mutation frequency was determined by DNA structure rather than UV-induced DNA adduct yield (3,52). Moreover, CPD removal is influenced not only by the DNA sequence at the site of adduct formation, but also by the surrounding DNA sequence (9).

Nucleosome rearrangement must occur in regions of DNA damage to allow the DNA repair complex to bind. Our laboratory has shown that CPD adducts destabilize DNA interactions with the histone octamer leading to a more open conformation (24,53), which could facilitate nucleosome remodeling. Interestingly, a chromatin remodeling complex that contains SNF2h, a member of the human ISWI family of chromatin remodeling factors, specifically associates with Alu-containing regions of human DNA (54). Snf2h binds transiently, but interactions are stabilized in the presence of replication foci in S-phase or UV-induced DNA damage (55). SNF2h rapidly accumulates at sites of UV damage within seconds of PCNA binding (56). In this study, we have shown that nucleosome occupancy at CPD hotspots decreases compared with the rest of the genome within 2 h of UV irradiation. Therefore, these hotspots of CPD formation are among the earliest sites associated with nucleosome rearrangement.

Our results represent the first chromosome scale map of UV-induced DNA lesions in the human genome, and reveal the sequence features and dynamic chromatin changes associated with CPD hotspots. We hypothesize that the identified hotspots of CPD formation occur because the sequence context and chromatin structure in these regions is conducive to DNA photoproduct formation. The ability to directly associate changes in nucleosome occupancy at sites of DNA lesions will prove to be a useful tool in correlating nucleosome level changes associated with efficiently repaired sites of DNA damage in the human genome.

## SUPPLEMENTARY DATA

Supplementary Data are available at NAR Online.

## FUNDING

The National Institute of Environmental Health Sciences, National Institutes of Health [ES002614 to M.J.S., reentry supplement to A.G.Z.]. Funding for open access charge: National Institutes of Health.

*Conflict of interest statement*. None declared.

## Supplementary Material

Supplementary Data
