# Supplementary material for: High-resolution characterization of CPD hotspot formation in human fibroblasts
Source: Nucleic Acids Res. 2013 Oct 16;42(2):893–905. doi: 10.1093/nar/gkt912 (PMC3902913; doi:10.1093/nar/gkt912)
Supplement: Supplementary Data [file supp_42_2_893__index.html]

High-resolution characterization of CPD hotspot formation in human fibroblasts — High-resolution characterization of CPD hotspot formation in human fibroblasts — Supplementary Data 

# High-resolution characterization of CPD hotspot formation in human fibroblasts

## Supplementary Data

files

**Files in this Data Supplement:**

- Supplementary Data - pdf file
