# Supplementary material for: High-resolution characterization of CPD hotspot formation in human fibroblasts
Source: Nucleic Acids Res. 2013 Oct 16;42(2):893–905. doi: 10.1093/nar/gkt912 (PMC3902913; doi:10.1093/nar/gkt912)

Supplementary Figure 1: CPD hotspots occur almost equally in genic and intergenic regions. CPD hotspot association with genomic regions was determined based on NCBIv36/hg18 RefSeq genome annotation.

Supplementary Figure 2: Approximately 20% of CPD hotspots are associated with more than one repeat element. Representative association of a CPD hotspot (green bars) associated with multiple repeat elements on chromosome 6. Hotspots were visualized using CisGenome software. Gaps indicate masked repeat regions which are not covered by probes.

Supplementary Figure 3: CPD hotspots are associated with repeat elements in both genic and intergenic regions approximately equally on both chromosomes. CPD hotspot association with genomic regions was determined based on NCBIv36/hg18 RefSeq genome annotation.

Supplementary Figure 4: CPD hotspots were confirmed using qPCR to amplify identified hotspots from chromosome A) 1 and B) 6 as well as a region with no identified hotspot on chromosome 1 (C). Orange bars indicate the regions amplified.

Supplementary Figure 5: Frequency of CPD Hotspots and random regions Compared to Alu Elements. (A) Chromosome 1 and (B) chromosome 6.

Supplementary Figure 6: CPD hotspots formed equally on the polydT and nonpolydT side of polydT Alu repeats. (A) Chromosome 1 and (B) chromosome 6. The apparent reduction in hotspot formation represented in the shaded regions corresponds to approximately one probe length (25 bp) from the edge of a masked repeat and is likely an artifact of the microarrays.

Supplementary Fig. 1

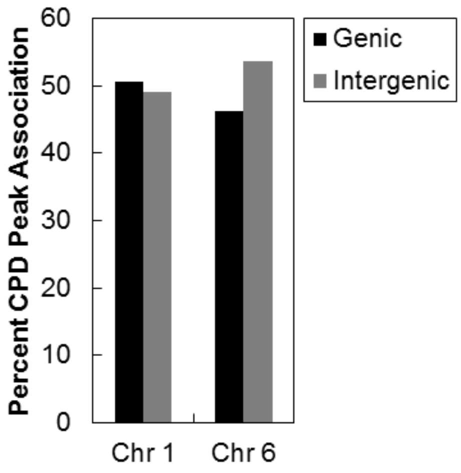

Supplementary Fig. 2

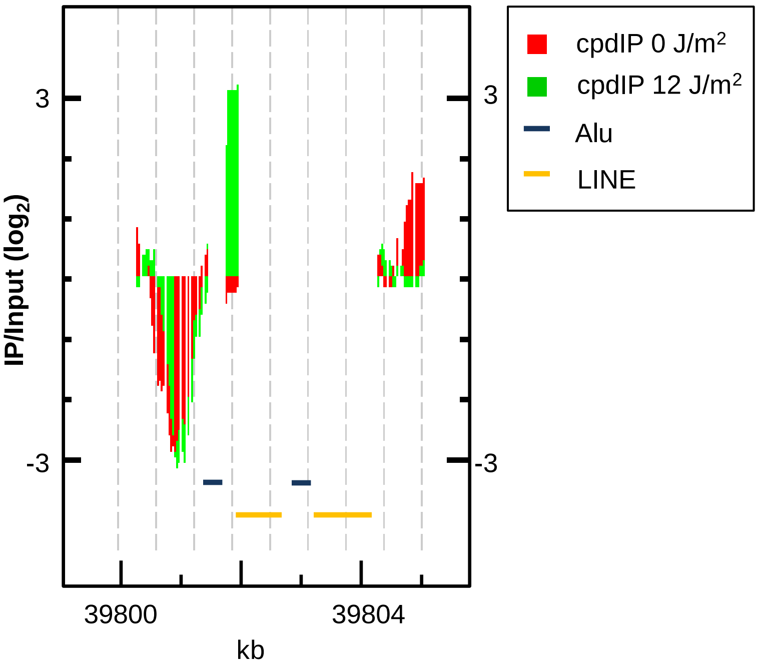

Supplementary Fig. 3

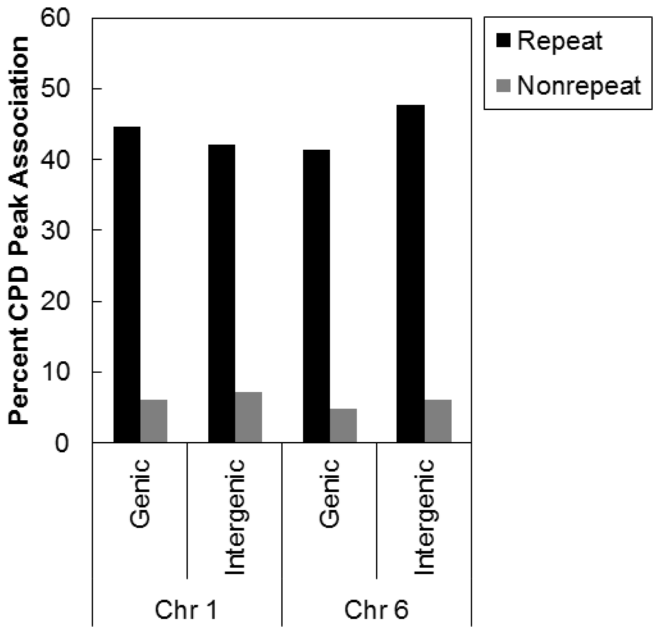

Supplementary Fig. 4

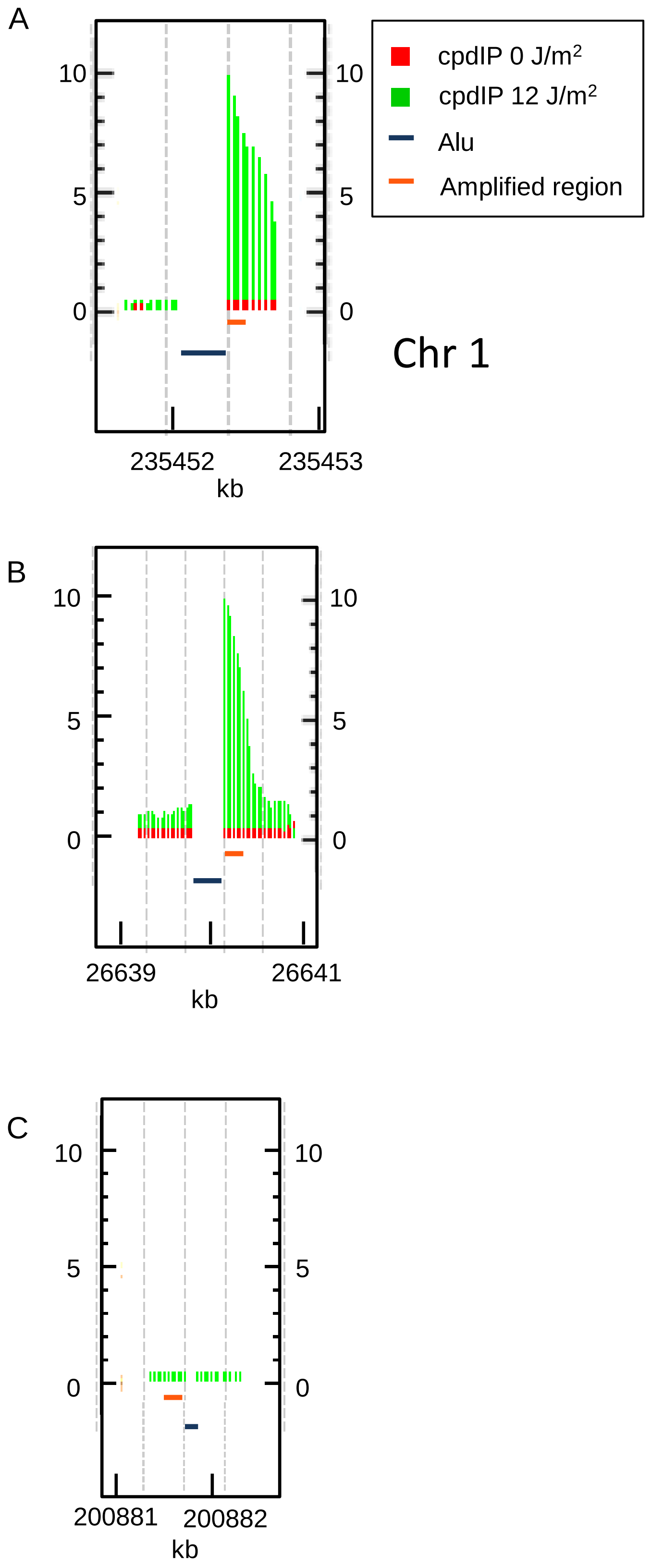

Supplementary Fig. 5

A

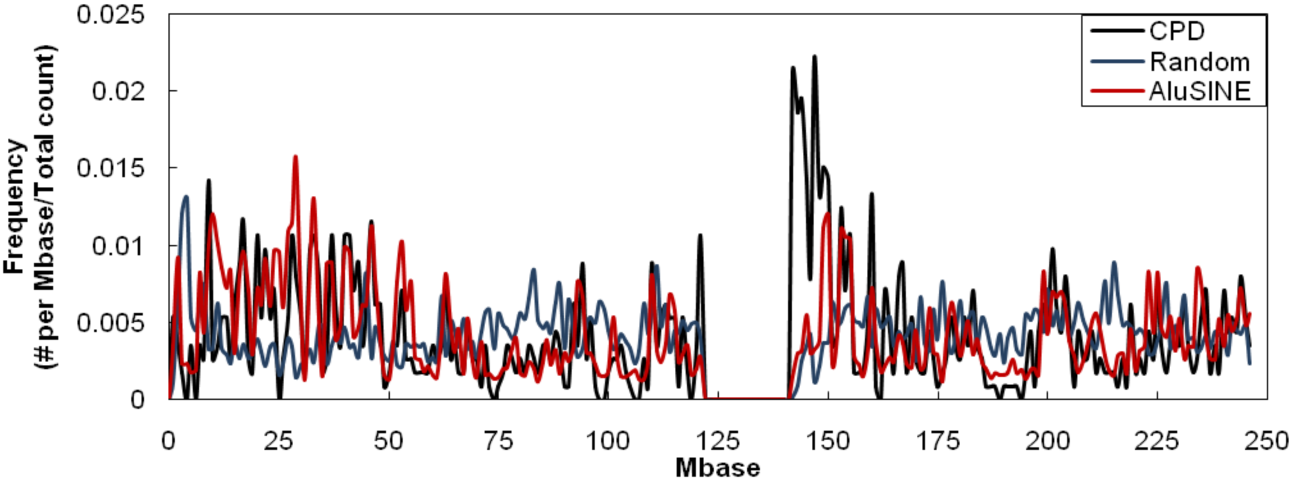

B

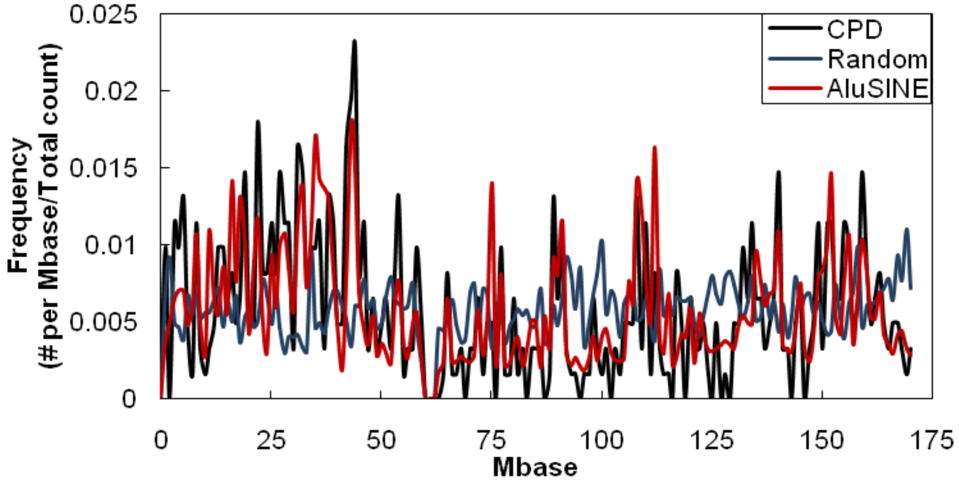

Supplementary Fig. 6

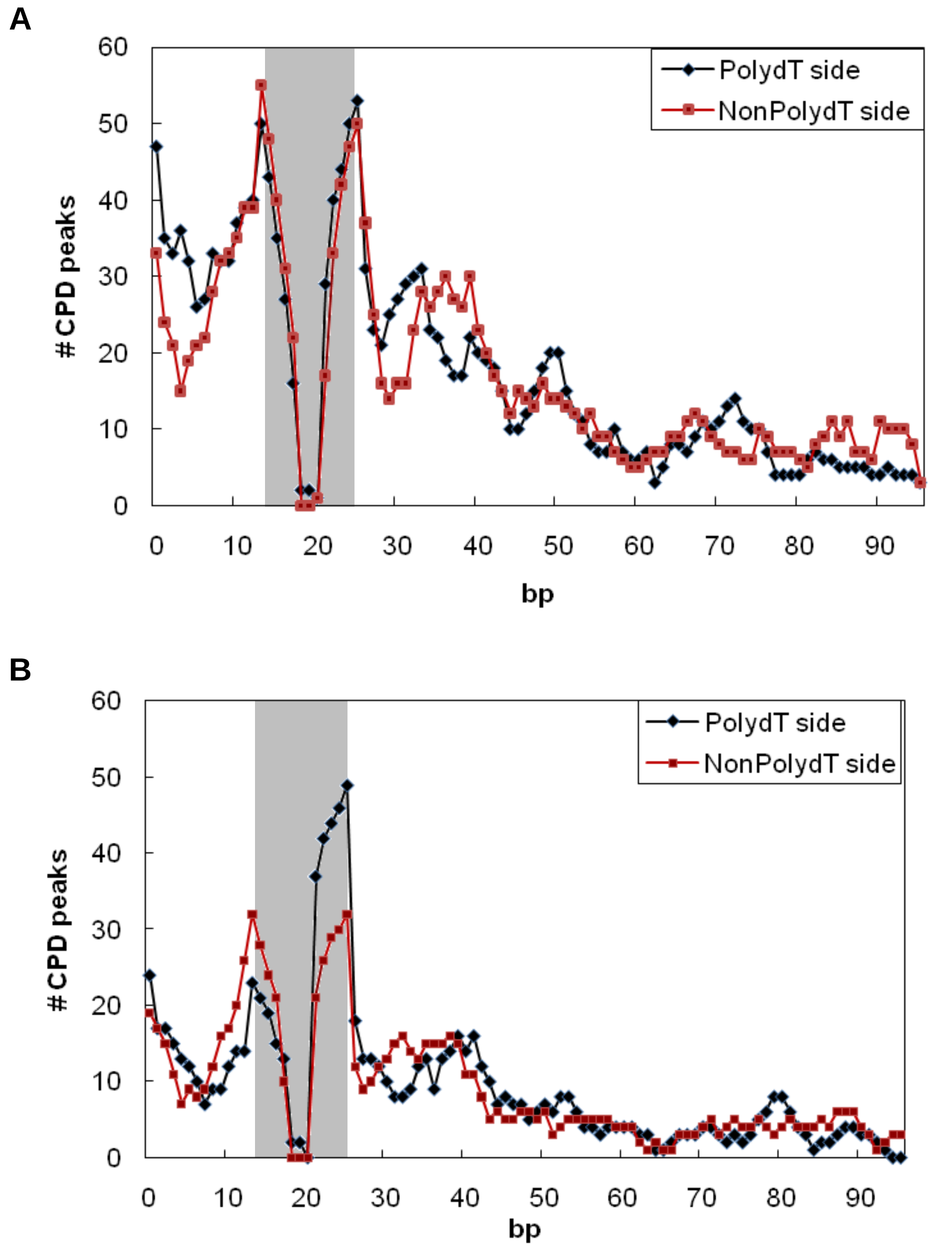

Supplement: Supplementary Data [file supp_gkt912_nar-02499-d-2013-File008.pdf]
